# Online pornography use during the COVID-19 pandemic: a review

**DOI:** 10.1192/j.eurpsy.2023.1650

**Published:** 2023-07-19

**Authors:** C. Portela, R. Dionísio, S. M. Sousa, M. Gonçalves

**Affiliations:** Hospital de Magalhães Lemos, Porto, Portugal

## Abstract

**Introduction:**

The Coronavirus (COVID-19) pandemic and the regulations enforced to control it caused significant alterations in daily routines worldwide. Lockdowns, remote working and schooling favoured virtual interactions and increased “free-time”, with the internet posing as a preferential means of distraction. Statistics from pornographic websites have shown a rise in traffic during lockdown periods, with problematic use of pornography (POPU) emerging as a potential mental health concern.

**Objectives:**

The authors aim to summarize current knowledge on the effects of the COVID-19 pandemic on online pornography use.

**Methods:**

Narrative review of articles referenced on PubMed and Google Scholar.

**Results:**

The increased exposure to the internet during the pandemic, combined with psychosocial factors such as social isolation, diminished physical contact and intimacy may have contributed to the reported surge in online pornography use. Other associated factors include emotional distress and less availability of other addictive substances and behaviours during confinement periods. Besides the spike in pornography consumption, other aspects were also affected, such as time of usage, search keywords and type of content, with an increase in engagement in illegal pornography. In susceptible individuals, these circumstances may lead to the development of POPU, characterized by impaired control, excessive time spent and perceived negative consequences. Currently, there is a lack of consensual diagnostic criteria for POPU, hindering the detection of these patients and timely management.

**Conclusions:**

Behavioural addictions are an emerging mental health problem, particularly the ones related to internet use. In the aftermath of the pandemic, considering the reported rise in online pornography use, an increase in POPU prevalence is expected. Therefore, more accurate and consensual diagnostic criteria are required, as well as a greater amount of evidence on the treatment of this disorder, in order to improve the approach to these patients.

**Disclosure of Interest:**

None Declared

